# Clinical Outcomes and Feasibility of Unilateral Biportal Endoscopic Decompression for Thoracic Ossification of the Ligamentum Flavum: A Retrospective Single-Center Study

**DOI:** 10.3390/jcm15197337

**Published:** 2026-09-22

**Authors:** Fangling Zhong, Chenxing Huang, Xiaoteng Feng, Xuru Feng, Zhaojun Cheng, Binwei Chen, Hui Ren, Weibo Yu, Xiaobing Jiang

**Affiliations:** Department of Spine Surgery, The Second Affiliated Hospital of Guangzhou Medical University, Guangzhou 510260, China

**Keywords:** unilateral biportal endoscopy, thoracic ossification of the ligamentum flavum, thoracic myelopathy, minimally invasive spine surgery, clinical efficacy

## Abstract

**Objective:** Thoracic ossification of the ligamentum flavum (T-OLF) is a primary cause of thoracic myelopathy in East Asian populations. Unilateral biportal endoscopic (UBE) decompression offers a minimally invasive alternative. This study evaluates the feasibility and clinical outcomes of UBE decompression in patients with symptomatic T-OLF. **Methods:** We retrospectively reviewed patients who previously underwent UBE decompression for symptomatic T-OLF between August 2023 and December 2025. Demographic data, operative details, and perioperative complications of the patients were recorded. Clinical outcomes were evaluated using the modified Japanese Orthopaedic Association (mJOA) score, American Spinal Injury Association (ASIA) score, Visual Analogue Scale (VAS), and MacNab criteria. The mJOA recovery rate (RR) was also calculated. **Results:** Fifty-one patients (mean age 65.1 ± 9.4 years) were included. The majority had single-level lesions (n = 44, 86.3%), which were predominantly located at the T10–11 level (n = 24/60 segments, 40.0%). The mean operation time per segment was 97.5 ± 24.4 min, and mean blood loss was 29.4 ± 14.7 mL. The mean mJOA score improved significantly from 6.6 ± 1.0 preoperatively to 9.4 ± 0.6 at final follow-up (*p* < 0.001), yielding a 63.5% RR. Based on MacNab criteria, 82.4% of patients achieved excellent or good outcomes. Perioperative complications included intraoperative dural tears in 5 patients (9.8%), postoperative cerebrospinal fluid leakage in 3 patients (5.9%), transient neurological deterioration in 2 patients (3.9%), and epidural hematoma in 1 patient (2.0%). All complications were managed successfully without permanent neurological sequelae. **Conclusions:** UBE decompression was associated with postoperative neurological improvement and acceptable perioperative outcomes in selected patients with symptomatic T-OLF. Further comparative studies are required to determine its relative benefits compared with other decompression techniques.

## 1. Introduction

The ligamentum flavum can undergo various pathological changes, including hypertrophy, calcification, cyst formation, and ossification [1]. Among these, thoracic ossification of the ligamentum flavum (T-OLF)—characterized by the replacement of normal ligamentous tissue with ectopic bone—is a leading cause of thoracic spinal stenosis and thoracic spondylotic myelopathy [2,3]. Epidemiological studies indicate a notably high prevalence of this condition in East Asian populations [4]. Large-scale imaging-based studies have revealed that the prevalence of TOLF ranges from approximately 3.8% detected by magnetic resonance imaging (MRI) to 21.8% identified by computed tomography (CT) [3,5]. Due to the insidious onset of T-OLF, its initial clinical symptoms are frequently confused with cervical or lumbar degenerative diseases, often leading to missed diagnoses or delayed interventions [6].

Once myelopathic symptoms emerge, conservative management is generally ineffective, rendering surgical decompression the mainstay of treatment [7]. Conventional open laminectomy provides direct neural access but is accompanied by significant drawbacks, including extensive paraspinal muscle disruption, prolonged postoperative axial pain, higher rates of incidental durotomy, and an increased risk of iatrogenic kyphosis [8]. These limitations have catalyzed a growing interest in minimally invasive endoscopic techniques designed to achieve equivalent neurological decompression while minimizing collateral tissue trauma [9].

Unilateral biportal endoscopy (UBE) has recently gained widespread popularity in lumbar spine surgery. This technique features independent viewing and working portals, offering excellent panoramic visualization and the unique ability to perform bilateral sublaminar decompression through a unilateral approach [10]. Although recent literature has demonstrated the safety and efficacy of UBE in treating T-OLF, relevant reports remain scarce and are limited by small sample sizes [11]. Therefore, the present study aims to report our institutional experience with UBE decompression for symptomatic T-OLF, with a specific focus on surgical techniques, perioperative safety, and neurological recovery.

## 2. Materials and Methods

### 2.1. Study Patients

This retrospective cohort study was approved by the Institutional Ethics Review Committee of The Second Affiliated Hospital of Guangzhou Medical University (Approval No. LYZX-2025-192-01, approval date: 14 November 2025). Between August 2023 and December 2025, a total of 75 patients diagnosed with symptomatic T-OLF were identified. Following eligibility assessment, 51 patients who underwent UBE decompression and completed the required clinical evaluation were included in the final analysis (Figure 1). All surgical procedures were performed by a single senior surgeon with over eight years of experience in UBE surgery. This study was conducted and reported according to the Strengthening the Reporting of Observational Studies in Epidemiology (STROBE) guidelines.

The inclusion criteria were as follows: (1) T-OLF confirmed by computed tomography (CT) and magnetic resonance imaging (MRI) with definitive signs of spinal cord compression; (2) progressive thoracic myelopathy or radiculopathy refractory to conservative management; and (3) UBE decompression performed at thoracic levels. Patients were excluded based on the following criteria: (1) previous thoracic spine surgery; (2) significant segmental instability or severe kyphosis necessitating synchronous instrumented fusion; (3) concurrent thoracic disc herniation or ossification of the posterior longitudinal ligament (OPLL) requiring an anterior approach; (4) severe cardiopulmonary comorbidities, coagulopathy, active systemic infection, or malignant tumors precluding general anesthesia; and (5) incomplete clinical or radiographic data. UBE was considered when patients had symptomatic thoracic myelopathy requiring posterior decompression but without severe instability, deformity, or lesions requiring anterior reconstruction.

### 2.2. Surgical Procedures

The patient was placed in a prone position under general anesthesia. Intraoperative neurophysiological monitoring, including somatosensory and motor evoked potentials, was routinely employed. The abdomen was freely suspended to minimize epidural venous congestion. Following fluoroscopic identification of the target level, two paramedian incisions (spaced 2–3 cm apart) were made approximately 1.5–2.0 cm lateral to the midline. The cranial incision typically served as the viewing portal, while the caudal incision functioned as the working portal. Continuous saline irrigation was established to maintain epidural hydrostatic pressure and a clear visual field. Soft tissues were meticulously cleared using a radiofrequency probe to expose the lamina and the interlaminar window. An ipsilateral laminotomy was performed utilizing a high-speed diamond burr and Kerrison rongeurs, strategically preserving the majority of the facet joint. For lesions with mild dural adhesion, a high-speed burr was initially used to rapidly remove the superficial lamina and cancellous bone, extending the resection to the cranial and caudal attachments of the ligamentum flavum. Subsequently, the ossified ligamentum flavum was adequately thinned using the burr. Finally, a nerve hook was utilized to carefully dissect the ventral surface of the ossified ligamentum flavum, and the ossified mass was gradually resected using Kerrison rongeurs. For lesions with severe dural adhesion or dural ossification (DO), an “eggshell” thinning technique was employed to avoid iatrogenic dural tears and spinal cord injury. Initially, a high-speed burr was used to drill the surrounding tissues, completely isolating the ossified mass from the adjacent structures. The ossified mass was then maximally thinned until translucent. This residual thin layer was then delicately detached and separated from the dura mater using a nerve hook. Ossified portions that were difficult to dissect safely were left in situ on the dural surface to form an “island.” This island would “float” dorsally along with the expansion of the dural sac, thereby achieving adequate decompression of the spinal cord. To achieve bilateral decompression, the endoscope was tilted to facilitate a contralateral “over-the-top” sublaminar approach, allowing for the complete resection of the contralateral T-OLF while preserving the midline spinous process and contralateral facet joint. Upon completion of the decompression, hemostasis was secured via a radiofrequency probe and topical hemostatic agents. A drainage tube was inserted through the working portal, and the incisions were closed in layers.

### 2.3. Outcome Measures

The primary outcome measure was neurological recovery, assessed using the modified Japanese Orthopaedic Association (mJOA) score for thoracic myelopathy (maximum 11 points). The neurological recovery rate (RR) was calculated as follows: RR = [(postoperative mJOA-preoperative mJOA)/(11-preoperative mJOA)] × 100% [12,13]. Neurological status was further stratified according to the American Spinal Injury Association (ASIA) impairment scale. Secondary clinical outcomes included the quantification of back pain and lower-extremity symptoms using the Visual Analog Scale (VAS, 0–10) and the evaluation of global surgical outcomes via the modified MacNab criteria. Neurological status and clinical outcomes were evaluated preoperatively, during the early postoperative period before discharge, and at the final follow-up. Systematic documentation was maintained for perioperative data, specifically operative duration, estimated intraoperative blood loss, length of hospital stay, and postoperative complications. For each patient, total operative time was divided by the number of decompressed segments to obtain a patient-level operative time per segment; these normalized values were then summarized as mean ± standard deviation (SD). Finally, independent radiographic evaluations using CT and MRI scans were performed by two experienced musculoskeletal radiologists. The OLF morphology was classified according to the Sato classification. Interobserver agreement for Sato classification was assessed using Cohen’s kappa coefficient. In cases of disagreement, the final classification was determined through consensus discussion between the two radiologists.

### 2.4. Statistical Analysis

Statistical analyses were performed using SPSS version 26.0. Continuous variables were presented as mean ± SD. The distribution of continuous variables was assessed using the Shapiro–Wilk test. Variables showing normal distributions were analyzed using paired *t*-tests, whereas non-normally distributed variables were analyzed using Wilcoxon signed-rank tests. The longitudinal changes in VAS scores and mJOA scores were evaluated by paired comparisons between preoperative and follow-up assessments. Categorical variables were analyzed using the chi-square test or Fisher’s exact test, as appropriate. Exploratory analyses of intraoperative dural tears were conducted at the decompressed-segment level. For 2 × 2 comparisons of radiological characteristics, conditional maximum-likelihood odds ratios (ORs), exact 95% confidence intervals (CIs), and two-sided *p* values were obtained using Fisher’s exact test. Each decompressed segment was treated as an analytic unit; within-patient correlation among segments from multilevel cases was not modeled. A two-sided *p* value < 0.05 was considered statistically significant.

## 3. Results

A total of 51 patients (27 males, 24 females; mean age 65.1 ± 9.4 years) who underwent UBE decompression for symptomatic T-OLF were included in this study. The mean body mass index (BMI) was 25.6 ± 4.1 kg/m^2^. Preoperative symptoms had been present for more than 12 months in 21 patients (41.2%), while 16 (31.4%) and 14 (27.5%) patients experienced symptoms for 6 to 12 months and less than 6 months, respectively. Preoperative comorbidities were frequently observed, including hypertension (n = 23, 45.1%), osteoporosis (n = 19, 37.3%), diabetes mellitus (n = 12, 23.5%), ischemic heart disease (n = 9, 17.6%), and gout (n = 3, 5.9%). The most prevalent clinical presentation was back pain/girdle sensation (n = 40, 78.4%), followed by sensory impairment, gait disturbance, and lower-extremity weakness (Table 1).

A total of 60 thoracic levels were decompressed across the 51 patients. Radiographically, the ossified lesions were predominantly located in the lower thoracic spine, most frequently at T10–11 (n = 24, 40.0%) and T11–12 (n = 16, 26.7%). A representative case of T10–11 OLF and its successful endoscopic management is shown in Figure 2. According to the Sato classification for OLF morphology, the extended (n = 17, 28.3%) and enlarged (n = 16, 26.7%) types were the most commonly encountered (Table 1). The interobserver agreement between the two radiologists for Sato classification was substantial (Cohen’s κ = 0.85, *p* < 0.001), indicating good reproducibility of the morphological assessment. All discrepant assessments were resolved by consensus review. The vast majority of the cohort underwent single-level decompression (n = 44, 86.3%), while two-level and three-level procedures were performed in 5 (9.8%) and 2 (3.9%) patients, respectively. Notably, a complex case involving three non-contiguous segments (T2–3, T6–7, and T10–11) was successfully managed through two staged UBE procedures (Figure 3). All UBE procedures were completed without conversion to open surgery. Although several cases involved technically challenging conditions, including severe dural adhesion, adequate decompression was achieved using the planned endoscopic approach without meeting predefined criteria for conversion. The mean operative time per decompressed segment, calculated at the patient level, was 97.5 ± 24.4 min, and the mean estimated intraoperative blood loss was 29.4 ± 14.7 mL. The average hospital stay was 9.8 ± 3.1 days. The mean duration of follow-up was 12.1 ± 6.6 months (range, 3–26 months) (Table 2). Six patients had follow-up durations of less than 6 months.

Perioperative complications occurred in a limited number of cases and were effectively managed without permanent sequelae (Table 2). Five cases (9.8%) of intraoperative dural tears were observed and promptly managed with the application of fibrin glue. Among the five patients with intraoperative dural tears, three developed postoperative cerebrospinal fluid (CSF) leakage, while the other two remained free of leakage after fibrin glue sealing. All CSF leakage cases resolved with conservative management, including strict bed rest and continuous drainage, and none required secondary dural repair. No hyperalgesia, head pain, or neck pain was found among patients with a dural tear. Transient neurological deterioration was observed in 2 patients (3.9%); both experienced complete spontaneous recovery during the follow-up period. Additionally, one patient (2.0%) developed a postoperative epidural hematoma that resolved conservatively. Notably, there were no instances of surgical site infection, permanent iatrogenic neurological deficits, or secondary segmental instability requiring subsequent fusion within the follow-up time frame.

Clinical and neurological functions demonstrated statistically significant improvements following UBE decompression (Table 3). The mean VAS score for back pain decreased substantially from 5.9 ± 1.6 preoperatively to 4.0 ± 1.2 immediately postoperatively, and further reduced to 1.6 ± 0.9 at the final follow-up. Similarly, the mean VAS score for lower-extremity symptoms improved from 6.1 ± 1.3 at baseline to 4.1 ± 1.1 postoperatively, reaching 2.1 ± 1.0 at the final assessment. Neurological function, quantified by the mJOA score, improved remarkably from a preoperative baseline of 6.6 ± 1.0 to 8.1 ± 0.7 postoperatively, achieving 9.4 ± 0.6 at the final follow-up (*p* < 0.001). Based on these mJOA scores, the mean neurological recovery rate (RR) achieved at the final assessment was 63.5%. This recovery was further corroborated by longitudinal improvements in the ASIA impairment scale: all 4 patients initially presenting with Grade C improved to Grade D, and among the 11 initial Grade D patients, 6 achieved normal motor and sensory function (Grade E). According to the modified MacNab criteria, global outcomes were rated as excellent in 28 patients (54.9%), good in 14 (27.5%), and fair in 9 (17.6%), resulting in a highly favorable excellent-to-good outcome rate of 82.4% (42/51), with no patients reporting worsened conditions. Importantly, neurological improvement was consistently observed across clinician-based assessments (mJOA and ASIA grades) and patient-reported outcomes (VAS and MacNab criteria), suggesting concordant functional recovery after surgery.

Because previous studies have suggested that specific radiological features may increase the risk of dural injury during T-OLF surgery, exploratory analyses were performed using segment-level data. Sato classification showed an association with intraoperative dural tears in this exploratory segment-based analysis (Fisher’s exact test, *p* = 0.037). Dural tears occurred more frequently in fused (33.3%) and tuberous (14.3%) lesions compared with lateral and extended types (Table 4). When fused and tuberous morphologies were grouped, dural tears occurred in 3 of 13 segments (23.1%) compared with 2 of 47 segments (4.3%) with other morphologies (conditional OR, 6.46; exact 95% CI, 0.65–86.89; *p* = 0.063). Comma sign-positive lesions also showed a higher point estimate (conditional OR, 7.95; exact 95% CI, 0.52–95.37; *p* = 0.074), although neither association reached statistical significance. The estimates were imprecise because only five dural-tear events occurred (Table 5).

## 4. Discussion

The primary objective of this retrospective study was to evaluate the feasibility and clinical outcomes of UBE decompression for thoracic myelopathy secondary to T-OLF. Our findings demonstrate that UBE decompression was associated with postoperative improvement in neurological function and pain-related outcomes in patients with symptomatic T-OLF. Neurological function, measured by the mJOA score, improved remarkably from a preoperative mean of 6.6 to 9.4 at the final follow-up. The average RR was 63.5%, which was comparable to that reported by Zhong et al. (65.6%) and higher than that reported by Gatam et al. (43.2%) [14,15]. Furthermore, 82.4% of patients reported excellent or good global clinical outcomes based on the modified MacNab criteria. These postoperative improvements, together with the observed perioperative complication profile, suggest that UBE is a feasible approach for selected patients with symptomatic T-OLF. Although validated MCID thresholds for the 11-point thoracic mJOA score are unavailable, the observed improvement, together with ASIA grade improvement, VAS reduction, and favorable MacNab outcomes, suggests neurological and symptomatic improvement after UBE decompression. However, these findings should be interpreted cautiously because of the lack of established MCID values and a control group.

Conventional open laminectomy remains an established surgical approach for thoracic spinal decompression [16]. However, thoracic decompression is technically challenging because of the relatively narrow spinal canal, physiological kyphotic alignment, and vulnerability of the thoracic spinal cord vascular supply [17]. Traditional posterior approaches may require wider exposure of posterior elements and paraspinal soft tissue dissection, which can potentially contribute to increased postoperative pain, greater tissue disruption, and alterations in spinal biomechanics in selected patients [18]. Therefore, minimally invasive techniques have been increasingly explored with the aim of achieving adequate neural decompression while reducing surgical invasiveness. By utilizing the natural interlaminar window, UBE adopts an “over-the-top” sublaminar approach, precisely preserving the midline posterior ligamentous complex, spinous processes, and the majority of the contralateral facet joints [19]. Previous studies have compared the clinical efficacy of UBE with traditional open thoracic laminectomy for the treatment of T-OLF. For instance, Gatam et al. demonstrated that the UBE technique facilitates faster decompression, significantly reducing both intraoperative blood loss and hospital length of stay [15]. Similarly, Deng et al. reported that applying UBE for T-OLF not only shortened operative time and hospitalization but also yielded highly satisfactory clinical outcomes [20]. The technical characteristics of UBE may facilitate adequate decompression while preserving posterior anatomical structures; however, whether these features translate into superior clinical outcomes requires further investigation.

Compared with other minimally invasive approaches, such as uniportal endoscopy and tubular decompression, UBE may offer technical advantages for selected T-OLF cases by providing independent viewing and working portals, which facilitate instrument maneuverability and contralateral decompression [21,22,23,24]. This feature may be particularly beneficial for complex lesion morphologies, including enlarged, fused, and tuberous Sato types, which accounted for a substantial proportion of our cases [25]. Continuous irrigation and the use of standard spinal instruments may further contribute to maintaining a clear operative field and facilitating controlled decompression [26]. However, whether these technical advantages translate into superior clinical outcomes requires further comparative studies.

Despite its minimally invasive nature, inherent surgical risks associated with T-OLF resection warrant careful management. Incidental durotomy and subsequent postoperative CSF leakage remain important complications during T-OLF surgery, particularly in cases with dural adhesion or dural ossification [27]. In our series, intraoperative dural tears occurred in 5 patients (9.8%), which is comparable to or lower than the rates reported in previous UBE studies and remains lower than the 15.8% incidence reported for conventional open surgery [17]. All dural defects were treated intraoperatively with fibrin glue sealing and meticulous multilayer closure. Although postoperative CSF leakage occurred in three patients, all cases resolved completely with conservative treatment, suggesting that prompt intraoperative management and careful wound closure may effectively mitigate the clinical consequences of this complication. Previous research indicates that the presence of the tram track sign and comma sign on axial CT, as well as the Sato classification (fused or nodular type), are significantly associated with an elevated risk of intraoperative dural tears [28,29]. Minor dural tears can typically be managed effectively with standard conservative protocols—including strict supine bed rest and patent local drainage—without necessitating primary surgical repair. Severe tears are best managed using a multilayered closure technique. This typically involves the precise application of an artificial dural patch and fibrin glue over the defect, followed by meticulous, water-tight suturing of the deep fascia and muscle layers to eliminate dead space [30]. Although transient neurological deterioration was observed in 2 patients (3.9%)—generally attributed to reperfusion injury of the chronically ischemic spinal cord or micro-concussive forces during bone resection—both patients achieved complete spontaneous recovery. Previous studies have reported complications associated with thoracic UBE. For instance, in a 16-patient cohort, Deng et al. reported spinal cord injury (n = 2), inadequate decompression (n = 1), excessive facet resection (n = 1), hematomas (n = 2), and delayed spinous process fracture (n = 1) [20]. Similarly, among 14 patients, Kim et al. documented CSF leakage with postural headache (n = 2), posterior neck pain (n = 2), and lower-extremity hyperalgesia (n = 2) [11]. Collectively, these findings emphasize that while the UBE approach presents a favorable perioperative complication profile and distinct advantages for T-OLF resection, mitigating the spectrum of potential complications requires meticulous surgical technique and vigilant perioperative care.

The resection of T-OLF remains technically demanding because severe dural adhesion and dural ossification are frequently encountered. Forceful avulsion or piecemeal removal of a densely ossified lesion may transmit excessive mechanical stress to the spinal cord and increase the risk of dural injury or neurological deterioration. To minimize these risks, we routinely adopted an “eggshell” thinning technique combined with the “floating” method. Specifically, the ossified mass was progressively drilled from the periphery toward the center with a high-speed diamond burr until it became extremely thin and translucent. The residual shell was then carefully separated from the dura with a nerve hook or micro-dissector, while portions with inseparable adhesion were intentionally left as a thin floating remnant on the dural surface to avoid aggressive manipulation. This stepwise decompression strategy helps redirect operative forces away from the spinal cord and may contribute to technical control during decompression, particularly in patients with marked dural adhesion or dural ossification. Therefore, regardless of whether an endoscopic or open approach is used, meticulous handling and strict adherence to a gentle decompression principle are essential during T-OLF surgery.

This study has several limitations. First, the retrospective single-center design and relatively small sample size may introduce selection bias and limit the generalizability of the findings. Second, all procedures were performed by a single experienced surgeon, which ensured procedural consistency but may limit applicability to surgeons with different levels of experience, particularly during the learning curve of thoracic UBE surgery. Third, although the follow-up period was sufficient for assessing early neurological recovery and symptom improvement, it was insufficient for evaluating long-term outcomes, including recurrence of ossification, delayed instability, deformity progression, and revision surgery. Moreover, six patients had follow-up durations of less than 6 months (minimum, 3 months); therefore, their final-follow-up mJOA and MacNab assessments primarily reflect early postoperative recovery and may not represent durable clinical benefit. Fourth, the absence of a comparative control group prevents definitive conclusions regarding the relative effectiveness of UBE compared with other decompression techniques. In addition, the exploratory dural-tear analyses were performed at the segment level, and seven patients contributed more than one segment. Within-patient correlation was not modeled, which may have underestimated statistical uncertainty. Because only five dural-tear events occurred and multiple comparisons were not formally adjusted, the corresponding effect estimates and *p* values should be interpreted cautiously. Future prospective multicenter comparative studies with larger cohorts and longer follow-up are warranted to further validate these findings.

In conclusion, UBE decompression was associated with neurological improvement and acceptable perioperative outcomes in selected patients with symptomatic T-OLF. Further multicenter prospective studies with longer follow-up are required to determine the durability of neurological improvement, recurrence patterns, and long-term structural outcomes after UBE decompression.

## Figures and Tables

**Figure 1:**
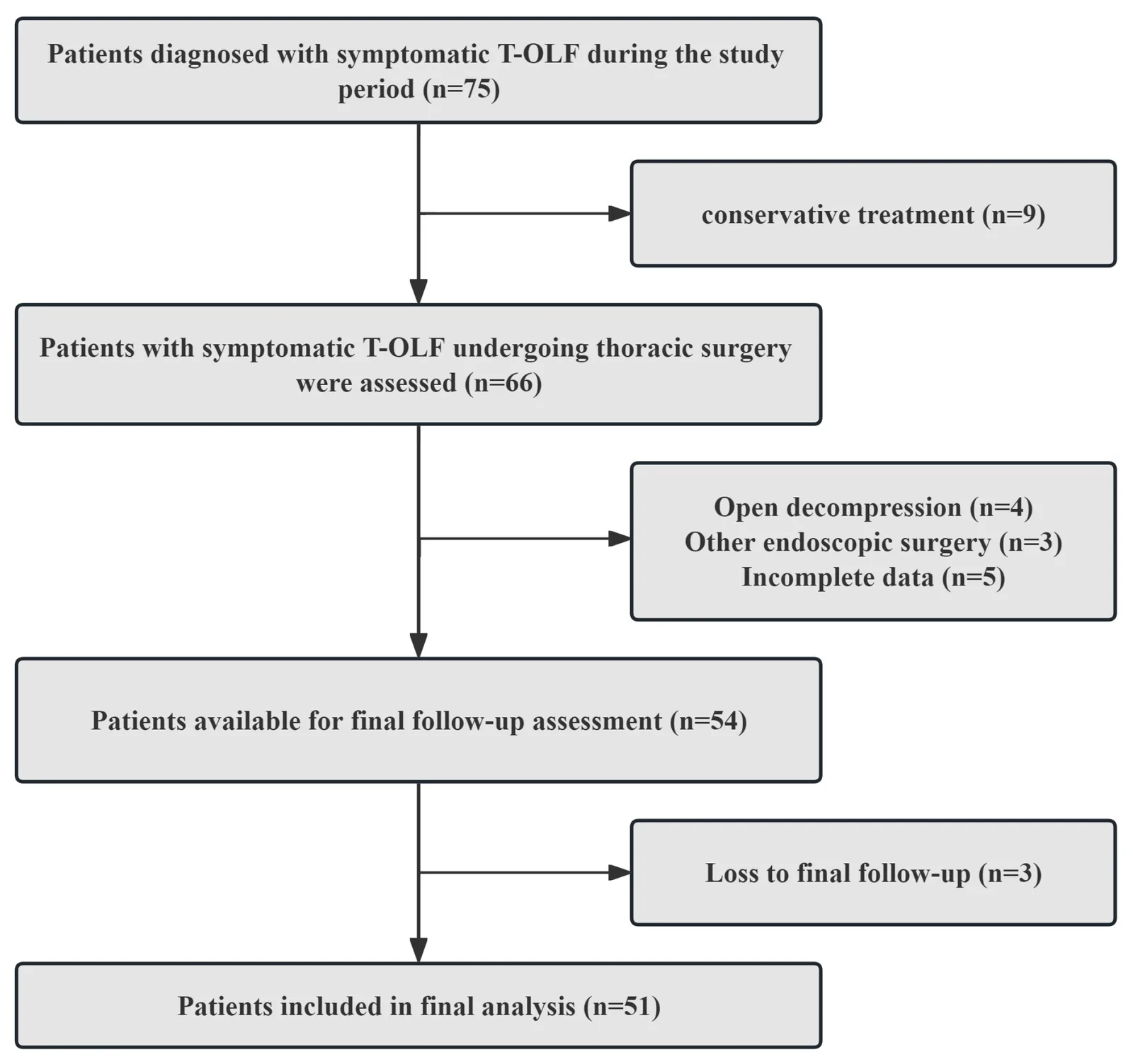
Flow diagram of patient selection.

**Figure 2:**
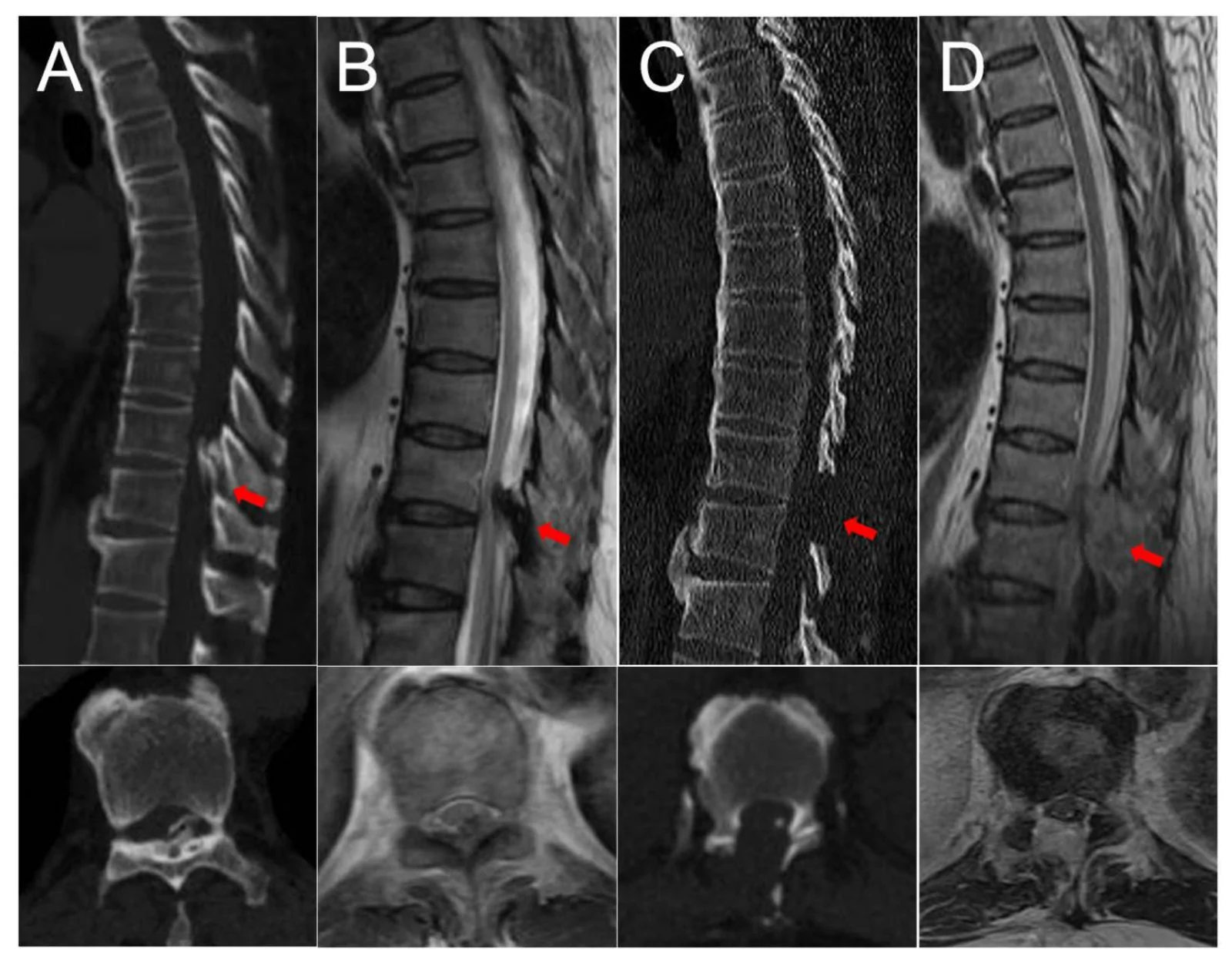
Preoperative and postoperative imaging of UBE decompression for T10–11 T-OLF. (**A**,**B**) Preoperative sagittal and axial CT images (**A**) and T2-weighted MRI scans (**B**) showing T10–11 T-OLF with severe spinal cord compression (red arrows). (**C**,**D**) Postoperative sagittal and axial CT images (**C**) and T2-weighted MRI scans (**D**) showing adequate decompression with removal of the compressive lesion and expansion of the dural sac (red arrows).

**Figure 3:**
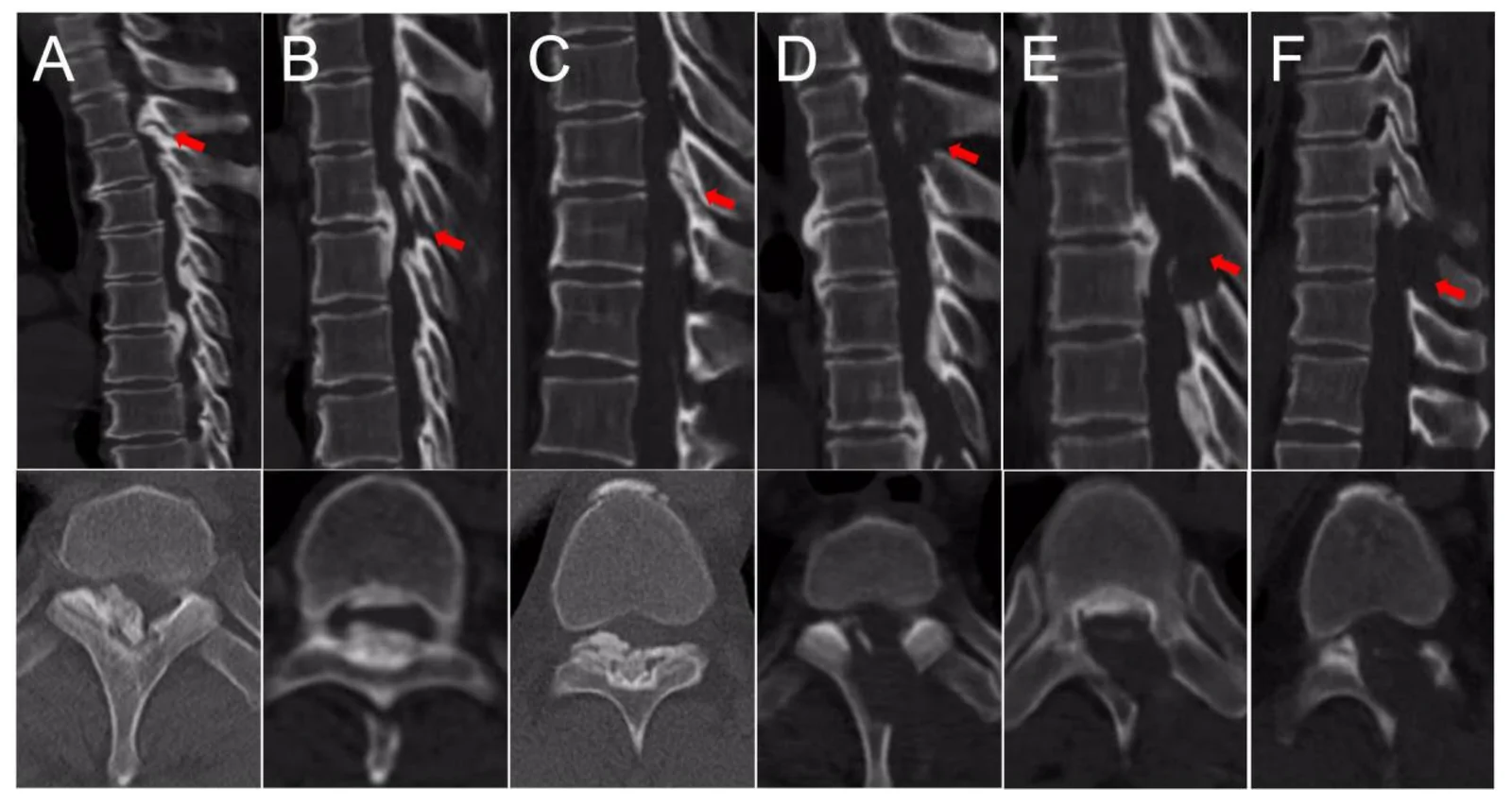
Staged UBE decompression for noncontiguous multi-level T-OLF. (**A**–**C**) Preoperative sagittal and axial CT images showing lesions at T2–3 (**A**), T6–7 (**B**), and T10–11 (**C**) (red arrows). (**D**–**F**) Postoperative CT images showing decompression at T2–3 and T10–11 after the first-stage surgery (**D**,**F**) and at T6–7 after the second-stage surgery (**E**), confirming adequate decompression at all symptomatic levels.

**Table 1:** Patient demographics and baseline characteristics.

Characteristic	Value
Sex, n (%)	
Male	27 (52.9)
Female	24 (47.1)
Age (yr), mean ± SD	65.1 ± 9.4
BMI (kg/m^2^), mean ± SD	25.6 ± 4.1
Symptom duration (months), n (%)	
<6	14 (27.5)
6–12	16 (31.4)
>12	21 (41.2)
Preoperative comorbidities, n (%)	
Hypertension	23 (45.1)
Osteoporosis	19 (37.3)
Diabetes mellitus	12 (23.5)
Ischemic heart disease	9 (17.6)
Gout	3 (5.9)
Preoperative symptoms, n (%)	
Back pain/girdle sensation	40 (78.4)
Sensory disturbance	18 (35.3)
Gait disturbance	18 (35.3)
Lower-extremity weakness	14 (27.5)
OLF levels (total 60 segments), n (%)	
T2–3	4 (6.7)
T3–4	2 (3.3)
T4–5	1 (1.7)
T6–7	2 (3.3)
T9–10	11 (18.3)
T10–11	24 (40.0)
T11–12	16 (26.7)
Sato classification (total 60 segments), n (%)	
Lateral type	14 (23.3)
Extended type	17 (28.3)
Enlarged type	16 (26.7)
Fused type	6 (10.0)
Tuberous type	7 (11.7)

SD, standard deviation; BMI, body mass index; OLF, ossification of the ligamentum flavum.

**Table 2:** Perioperative parameters and surgical complications.

Characteristic	Value
Operating segments, n (%)	
Single level	44 (86.3)
2 Levels	5 (9.8)
3 Levels	2 (3.9)
Operating time per segment (min), mean ± SD	97.5 ± 24.4
Blood loss (ml), mean ± SD	29.4 ± 14.7
Hospital stays (days), mean ± SD	9.8 ± 3.1
Follow-up duration (months), mean ± SD	12.1 ± 6.6
Complications, n (%)	
Intraoperative dural tears	5 (9.8)
Postoperative CSF leakage	3 (5.9)
Transient neurological deterioration	2 (3.9)
Epidural hematoma	1 (2.0)

SD, standard deviation. Operative time per decompressed segment was calculated for each patient by dividing total operative time by the number of decompressed segments.

**Table 3:** Comparison of preoperative and postoperative clinical outcomes.

Variable	Preoperative	Postoperative	Final Follow-Up	Effect Size	95% CI
VAS for back pain	5.9 ± 1.6	4.0 ± 1.2 **	1.6 ± 0.9 **	−3.09	−3.37 to −2.81
VAS for lower-extremity symptoms	6.1 ± 1.3	4.1 ± 1.1 **	2.1 ± 1.0 **	−3.02	−3.30 to −2.74
mJOA score	6.6 ± 1.0	8.1 ± 0.7 **	9.4 ± 0.6 **	2.77	2.49 to 3.05
Recovery rate (%)	-	-	63.5		
ASIA impairment scale, n (%)					
Grade C	4 (7.8)	1 (2.0)	0 (0.0)		
Grade D	11 (21.6)	10 (19.6)	9 (17.6)		
Grade E	36 (70.6)	40 (78.4)	42 (82.4)		
MacNab criteria, n (%)					
Excellent	-	-	28 (54.9)		
Good	-	-	14 (27.5)		
Fair	-	-	9 (17.6)		
Poor	-	-	0 (0.0)		

SD, standard deviation; CI, confidence interval; VAS, visual analogue scale; mJOA, modified Japanese Orthopaedic Association; ASIA, American Spinal Injury Association. ** Compared with the preoperative value, *p* < 0.001.

**Table 4:** Association between Sato classification and intraoperative dural tears.

Sato Classification	Total Segments, n	No Dural Tear, n (%)	Dural Tear, n (%)
Lateral type	14	14 (100.0)	0 (0.0)
Extended type	17	17 (100.0)	0 (0.0)
Enlarged type	16	14 (87.5)	2 (12.5)
Fused type	6	4 (66.7)	2 (33.3)
Tuberous type	7	6 (85.7)	1 (14.3)
Total	60	55 (91.7)	5 (8.3)

**Table 5:** Association between radiological characteristics and intraoperative dural tears.

Variable	No Dural Tear (n = 55)	Dural Tear (n = 5)	OR (95%CI)	*p* Value
Fused/tuberous morphology	10	3	6.46 (0.65–86.89)	0.063
Tram-track sign	10	1	1.12 (0.02–13.10)	1.000
Comma sign	4	2	7.95 (0.52–95.37)	0.074
Dural ossification	14	3	4.26 (0.44–56.01)	0.132

OR, odds ratio; CI, confidence interval. ORs are conditional maximum-likelihood estimates. Exact 95% CIs and two-sided *p* values were calculated using Fisher’s exact test. For each variable, absence of the radiological characteristic was the reference category. Analyses were conducted at the decompressed-segment level.

## Data Availability

The datasets used and/or analysed during the current study are available from the corresponding author on reasonable request.
